# Poor uptake of an online intervention in a cluster randomised controlled trial of online diabetes education for rural general practitioners

**DOI:** 10.1186/s13063-017-1869-8

**Published:** 2017-03-23

**Authors:** Christine L. Paul, Leon Piterman, Jonathan E. Shaw, Catherine Kirby, Kristy L. Forshaw, Jennifer Robinson, Isaraporn Thepwongsa, Robert W. Sanson-Fisher

**Affiliations:** 1 Hunter Medical Research Institute, New Lambton Heights, Newcastle, NSW Australia; 2 Monash University, School of Rural Health, Churchill, VIC Australia; 3 Eastern Victoria General Practice Training, Churchill, VIC Australia; 4 Baker IDI Heart and Diabetes Institute, Clinical Diabetes and Epidemiology Group, Melbourne, VIC Australia; 5 Department of Epidemiology and Preventive Medicine, Monash University, Melbourne, VIC Australia; 6 University of Newcastle, School of Medicine and Public Health, Callaghan, NSW Australia; 7 Department of Community Medicine, Faculty of Medicine, Khon Kaen University, Khon Kaen, Thailand; 8 W4 HMRI Building, School of Medicine and Public Health, University of Newcastle, Callaghan, NSW 2308 Australia

**Keywords:** Type 2 diabetes, General practice, Online education, Medical education, Rural medicine

## Abstract

**Background:**

In Australia, rural and remote communities have high rates of diabetes-related death and hospitalisation. General practitioners (GPs) play a major role in diabetes detection and management. Education of GPs could optimise diabetes management and improve patient outcomes at a population level. The study aimed to describe the uptake of a continuing medical education intervention for rural GPs and its impact on the viability of a cluster randomised controlled trial of the effects of continuing medical education on whole-town diabetes monitoring and control.

**Method:**

Trial design: the cluster randomised controlled trial involved towns as the unit of allocation and analysis with outcomes assessed by de-identified pathology data (not reported here). The intervention programme consisted of an online active learning module, direct electronic access to specialist advice and performance feedback. Multiple rounds of invitation were used to engage GPs with the online intervention content. Evidence-based strategies (e.g. pre-notification, rewards, incentives) were incorporated into the invitations to enrol in the programme. Recruitment to the programme was electronically monitored through the hosting software package during the study intervention period.

**Results:**

Eleven matched pairs of towns were included in the study. There were 146 GPs in the 11 intervention towns, of whom 34 (23.3%) enrolled in the programme, and 8 (5.5%) completed the online learning module. No town had more than 10% of the resident GPs complete the learning module. There were no contacts made by GPs regarding requests for specialist advice. Consequently, the trial was discontinued.

**Conclusion:**

There is an ongoing need to engage primary care physicians in improving diabetes monitoring and management in rural areas. Online training options, while notionally attractive and accessible, are not likely to have high levels of uptake, even when evidence-based recruitment strategies are implemented.

**Trial registration:**

Australian New Zealand Clinical Trials Registry, identifier: ACTRN12611000553976. Retrospectively registered on 31 May 2011.

## Background

Type 2 diabetes is a mainly preventable chronic disease affecting an estimated 1 in 11 people worldwide, and this figure is rising [1]. Type 2 diabetes increases the risk of cardiovascular disease, stroke, blindness and limb amputations [1, 2]. The direct and indirect costs of diabetes and its associated complications are substantial, and the impact on individuals, families and national health systems can be burdensome. Early diagnosis and good control of type 2 diabetes with close monitoring of metabolic markers (blood glucose, blood lipids, blood glycosylated haemoglobin (HbA1c) and urinary albumin) and other cardiovascular disease indicators, such as blood pressure, can reduce the risk of diabetes complications [1, 3–6].

Primary care physicians, or general practitioners (GPs) in Australia, play a major role in diabetes detection and management. National guidelines specify the frequency of testing and cut-off points for optimal diabetes management in general practice [7]. However, achieving this at a population level remains challenging, with only just over 20% of Australian people receiving optimal management of their diabetes in 2011–2012 [8].

In Australia, rural and remote communities are at particular disadvantage with regard to diabetes care given that diabetes prevalence and rates of diabetes-related death, care activity and hospitalisation rise with increasing remoteness of residence [2, 9–11]. The proportion of diabetic patients meeting targets for total cholesterol, triglycerides and blood pressure levels has also been shown to be lower in rural areas compared with urban areas [12]. A recent study found that 60.5% of patients did not undergo the recommended 6-monthly HbA1c tests, and 34.1% did not undergo the recommended annual lipid testing. For those with at least one out-of-range test result, 79% of patients failed to receive a follow-up HbA1c test within the recommended 3 months [13]. Although people living outside major cities have less access to GP services overall, and very limited access to specialist services [14], there have been few studies investigating whether specific diabetes management training for rural-based GPs could improve patient outcomes at a population level.

Continuing medical education (CME) is fundamental to improving clinical care in general practice for diabetes and other conditions. Web-based or online CME is increasing in popularity and is of particular relevance to GPs in rural locations [15] where face-to-face training opportunities can be less flexible and accessible. A survey of rural GPs in Australia, found that while only 28.9% of GPs had used structured online learning for type 2 diabetes education in the prior 3 years, 49.0% expected to use this form of learning in the future with regard to diabetes care [16].

In order to examine the population-level effects of CME it is important to go beyond looking at samples of patients and providers who have consented to participate in a study, particularly given that recruitment of GPs can be highly challenging and yield low participation rates [17–20]. Capturing the whole patient population is necessary to provide generalisable data about the effectiveness of CME. This cluster randomised trial commenced in 2010, and aimed to use objective administrative data to examine the effectiveness of online diabetes CME and additional intervention strategies at the population level in the rural setting. To meet this objective, the study design used communities as the unit of analysis and administrative data sets (whole town de-identified pathology data). The centerpiece of the intervention was an online Active Learning Module (ALM), a structured learning tool for which GPs received continuing professional development points via their professional body. The protocol for this trial has been published [21]. This paper will describe the uptake of the CME intervention and its impact on the viability of the trial.

## Method

### Design and sample

The cluster randomised controlled trial involved towns as the unit of allocation and analysis. Towns were eligible for selection if they: had an ‘Australian Remoteness Index for Areas Plus’ (ARIA+) [22] classification of 2.0 or greater; had a population of 10,000 to 30,000 people; were in the Australian states of Victoria, New South Wales (NSW), or Queensland and had five or more full-time equivalent GPs. Eligible towns were matched in pairs within each state on the above variables, proportion of population identified as Indigenous, and socioeconomic status. Towns were randomised on a one-to-one allocation ratio via a computer-generated, stratified randomisation scheme in SAS (Statistical Analysis Software). Allocation remained concealed to all participants and all those assessing outcomes throughout the study. The selection, matching and randomisation of towns, and sample size calculation is described in more detail in the study protocol [21]. In the intervention towns, the intervention was offered to all listed GPs found to be practising in that town. Participation in the intervention did not require consent to trial participation.

### Intervention content

The intervention was offered over 2 years (mid-2011 to mid-2013) and designed to provide GPs with prerequisite knowledge for optimal primary care management of diabetes and opportunities to practice and refine skills in the practice setting. There were no restrictions on GPs’ access to other available forms of diabetes information and education opportunities during the period of the study. The intervention components were:

1. *Online ALM*: because multimedia education is more effective than a single medium [23], the ALM included a range of features and presentation types including: evidence-based Australian clinical guidelines, video demonstrations, case studies, knowledge-based quizzes, clinical audit, self-reflection activities and a moderated peer discussion forum. The ALM comprised approximately 6 h of learning activity, for which GPs received continuing professional development points via their professional body. The ALM was able to be accessed via password on any Internet-enabled device, at any time convenient to the GP. Sections of the ALM could be completed separately, and could be saved and completed at a later time. However, professional development points could only be awarded if the whole ALM was completed. Content for the ALM was developed in consultation with rural GPs and diabetes specialists to ensure accuracy and relevance to the rural GP population. The online ALM was consumer-tested by a small sample of four GPs and diabetes specialists to ensure that the final programme was user-friendly, accurate, and relevant to GPs. When a GP registered with the ALM, and email alert was sent to the research team as part of the hosting software package
2. *Access to specialist advice*: given that rural GPs may not have access to a diabetes specialist in their town, every invitation to promote the ALM also promoted the availability of an online request form for specialist advice regarding diabetes, accessed by secure log-in via the ALM. A diabetes specialist (medical doctor with specialist qualifications in endocrinology) was assigned to manage queries from GPs in the intervention towns. A number of diabetes specialists were made available on an ‘on-call’ basis over the duration of the study. This enabled GPs to access advice on applying the diabetes management principles learned in the educational programme in their day-to-day practice, and to ask for advice on more complex cases that arose. Importantly, this represents a mechanism for case-based learning, which has been demonstrated to be one of the most promising forms of CME [23]
3. *Town-based performance feedback*: at invitation rounds 3, 4, 5 and 6 GPs were automatically provided with town-based feedback which contained de-identified information about the proportion of diabetic patients in the town receiving testing at the recommended frequency and within target guidelines for HbA1c and blood lipids. Systematic reviews show that audit and feedback can have modest effects on changing provider practice [24]
4. *Comparative feedback*: at invitation rounds 3, 4, 5 and 6 GPs were automatically provided with a summary of the range of performance per GP in their town. This takes the form of a graph showing the proportion of patients per GP who meet guidelines for test frequency and management (meeting HbA1c targets). Comparative feedback has been shown to be an effective mean of changing surgeons’ behaviour [25, 26]. Both forms of feedback were derived from de-identified data provided by local pathology laboratories

### Control group

No interventions by the research team were being provided to this group. There were no restrictions on GPs’ access to other available forms of diabetes information and education opportunities during the period of the study.

### Recruitment of GPs to intervention components

**Fig. 1:**
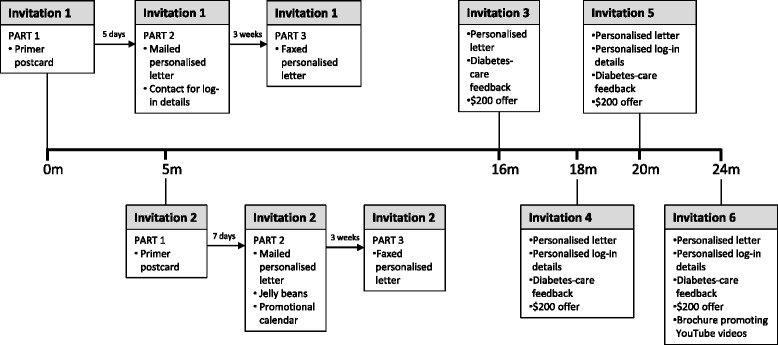
Timeline of general practitioner (GP) recruitment process over 24 months. All identified GPs in each town were sent invitations to participate at each invitation round

The availability of the ALM, specialist advice and feedback to GPs was advertised in local GP network newsletters in intervention towns. Invitation letters were also sent to every identified GP in these towns using a nationwide online medical database (the Medical Directory of Australia) and the Electronic Telephone Directory (Yellow Pages). Six rounds of invitations, with a total of 10 occasions of contact (Fig. 1), were completed over the 24-month intervention period. The invitations incorporated evidence-based strategies known to increase recruitment to research [27–31], including being authored by nationally recognised leaders in diabetes and primary care (authors JS and LP). In Australia, the recognised professional development programme for GPs (for which this ALM was eligible) takes place in designated trienniums, covering the 3 years of total time that GPs have to accumulate professional development points. The invitation rounds in this study were timed to cross over the final period of one triennium and the commencement of another triennium in order to maximise the range of times and reasons for choosing to complete learning activities. See Fig. 1 for a timeline of the recruitment process.

### Data collection

Recruitment to the intervention online ALM was monitored during the intervention period. The number of GPs taking up the offer of the online learning module was assessed directly from use of the link to register for the module. Module completion was also obtained electronically via website-generated notification of when a participant had completed the final survey/self-assessment items which were part of the ALM. Use of the online access to specialist advice was also assessed using a log of contacts received.

### Statistical methods

Number of enrolments to the ALM and/or use of the expert advice service are provided.

## Results

### Sample

A total of 22 towns were eligible for the study (11 matched pairs), all of which were in NSW and Queensland. Analysis of the pathology data indicated 13,805 potential diabetes cases in the intervention sample (range = 121–1296 per town). A total of 146 GPs were identified in the 11 intervention towns at the close of the study (median = 13 per town; range = 5–28).

### Recruitment to intervention

- *ALM recruitment*: Table 1 shows the number of GPs who enrolled in the diabetes ALM at each recruitment period. The total of 34 enrolments represents 23.3% of the total number of GPs who could have enrolled in the ALM from each town. There was at least one GP in each town who enrolled in the ALM. In only one town was there more than one quarter of GPs enrolled in the ALM.

**Table 1:** Number of general practitioners (GPs) enrolling in the Active Learning Module (ALM) at each round of invitation (from identified *n* = 146)

Invitation round	Intervention month	Invitation content	GPs enrolling (*n*)
1	1	a) Primer postcard then b) mailed letter Faxed letter	a) 9 b) 5
2	5	a) Primer postcard then b) mailed letter with jellybeans and calendar c) Faxed letter	a) 3 c) 2
3	16	Mailed letter, ALM brochure, feedback and A$200 voucher offer	4
4	18	Mailed letter, ALM brochure, feedback, A$200 voucher offer and self log-in	8
5	20	Mailed letter, ALM brochure, feedback, A$200 voucher offer and self log-in	2
6	24	Mailed letter, ALM brochure, feedback, A$200 voucher offer, self log-in, YouTube video brochure	1
Total enrolment			34 (23.3%)
- *ALM completion*: a total of 8 GPs (5.5%) completed the ALM. Six of the 11 towns had one or more GPs who completed the ALM, with no town having more than 10% of their GPs complete the ALM. No GPs from the control group towns enrolled in the ALM.
- *Specialist advice*: there were no contacts made regarding requests for specialist advice.

### Impact of recruitment on cluster randomised controlled trial

Given the small number of GPs engaging with the intervention activities, in that 23% of GPs enrolled and 5% of GPs completed the ALM, it was decided that there was no likelihood of the intervention having a detectable positive impact on the main trial outcome – i.e. the proportion of diabetes patients who received regular HbA1c tests and who exhibited appropriate glycaemic control. Therefore, it was agreed by the study investigators that the trial would be discontinued.

## Discussion

This study describes the extensive efforts made to recruit rural GPs to an online education and support initiative regarding improving diabetes care in the primary care setting. Given that the number of GPs completing the ALM was too few to expect an intervention effect, the intervention trial was discontinued, that is, no follow-up data were collected.

Given that a similar study by this lead author has shown similar difficulties in recruiting GPs to online learning [20], it is perhaps unsurprising that the trial was not successful. In fact, a number of studies have experienced difficulty in the first step of recruiting GPs to participate in online education [18, 32].

Even though our recruitment strategy was evidence-based [27–31], with quite substantial incentives (e.g. a voucher for A$200) and a large number of contacts (10 contacts over six time periods), this resulted in less than one quarter of GPs showing interest in the ALM. More intensive recruitment methods, such as face-to-face contact, in person or by telephone, may have yielded better participation rates; however, this is known to be labour-intensive and cost-prohibitive [33].

A question that arises from the failure of the study was whether GPs thought that their current knowledge was sufficient without further education. This seems unlikely in that a survey conducted on a very similar population of rural GPs [16] during a similar time period found that only half felt up-to-date with new technology and treatment for type 2 diabetes, although most felt confident in their ability to manage most aspects of diabetes care.

Despite the poor uptake of the intervention, the baseline data for the study has identified an ongoing need for improvement in the rates of diabetes monitoring and management in rural and remote areas of Australia [13].

### Limitations

A major limitation of this study was a lack of data regarding the availability and uptake of other online or face-to-face learning opportunities for GPs during the study period. While very few ALMs were evident at the commencement of the study, there are now a number of online diabetes care courses for GPs available from various recognised organisations in Australia including the Royal Australian College of General Practitioners (RACGP). Unfortunately, the RACGP is not able to disclose the number of GPs who have undertaken their training courses on diabetes. It is possible that the GPs contacted for this study may have completed other face-to-face or online courses during the period of the study. In addition, as the intervals between invitation rounds were of varying length, and the enrolment numbers were so small, it is difficult to identify whether any particular recruitment method was more effective than another. Another limitation of the study was not collecting feedback from the physicians contacted to be part of the study. This feedback may have included information on time constraints, acceptability of the offered intervention, current education on diabetes management and the current availability of access to diabetes specialists. However, given response rates to such a survey would be low, such an approach may not have provided useful data.

## Conclusions

There is a need to engage primary care physicians in improving diabetes monitoring and management in rural areas. Online training options, while notionally attractive and accessible, are not likely to have high levels of uptake, even when evidence-based recruitment strategies are implemented.

### Lessons learned from conducting the trial

The results of the trial indicated that even with relatively intensive efforts, the recruitment of GPs to educational interventions is highly challenging. Online interventions, although notionally convenient and accessible, were shown to be easily ‘ignorable’ in that a low proportion of the participants completed the learning module.

## Abbreviations

ALM: Active Learning Module
ARIA+: Australian Remoteness Index for Areas Plus
CME: Continuing Medical Education
GP: General practitioner
HbA1c: Glycosylated haemoglobin
NSW: New South Wales
RACGP: Royal Australian College for General Practitioners
SAS: Statistical Analysis Software
